# Reproducibility and Reliability of Quantitative and Weighted T_1_ and T_2_^∗^ Mapping for Myelin-Based Cortical Parcellation at 7 Tesla

**DOI:** 10.3389/fnana.2016.00112

**Published:** 2016-11-18

**Authors:** Roy A. M. Haast, Dimo Ivanov, Elia Formisano, Kâmil Uludaǧ

**Affiliations:** Department of Cognitive Neuroscience, Faculty of Psychology and Neuroscience, Maastricht UniversityMaastricht, Netherlands

**Keywords:** myelin-related cortical mapping, ultra-high-field MRI, quantitative MRI, MR parameters, anatomy

## Abstract

Different magnetic resonance (MR) parameters, such as R_1_ (=1/T_1_) or T_2_^∗^, have been used to visualize non-invasively the myelin distribution across the cortical sheet. Myelin contrast is consistently enhanced in the primary sensory and some higher order cortical areas (such as MT or the cingulate cortex), which renders it suitable for subject-specific anatomical cortical parcellation. However, no systematic comparison has been performed between the previously proposed MR parameters, i.e., the longitudinal and transversal relaxation values (or their ratios), for myelin mapping at 7 Tesla. In addition, usually these MR parameters are acquired in a non-quantitative manner (“weighted” parameters). Here, we evaluated the differences in ‘parcellability,’ contrast-to-noise ratio (CNR) and inter- and intra-subject variability and reproducibility, respectively, between high-resolution cortical surface maps based on these weighted MR parameters and their quantitative counterparts in ten healthy subjects. All parameters were obtained in a similar acquisition time and possible transmit- or receive-biases were removed during post-processing. It was found that CNR per unit time and parcellability were lower for the transversal compared to the longitudinal relaxation parameters. Further, quantitative R_1_ was characterized by the lowest inter- and intra-subject coefficient of variation (5.53 and 1.63%, respectively), making R_1_ a better parameter to map the myelin distribution compared to the other parameters. Moreover, quantitative MRI approaches offer the advantage of absolute rather than relative characterization of the underlying biochemical composition of the tissue, allowing more reliable comparison within subjects and between healthy subjects and patients. Finally, we explored two parcellation methods (thresholding the MR parameter values vs. surface gradients of these values) to determine areal borders based on the cortical surface pattern. It is shown that both methods are partially observer-dependent, needing manual interaction (i.e., choice of threshold or connecting high gradient values) to provide unambiguous borders.

## Introduction

The brain can be partitioned into distinct functional and anatomical areas based on functional specificity and histological markers, such as cyto-architecture, receptor-architecture and cortical myelin distribution (Brodmann, 1909; Zilles and Amunts, 2009; Nieuwenhuys et al., 2014). In particular, the distribution of cortical myelin (i.e., myeloarchitecture) is highly suitable for parcellating the brain (Brodmann, 1909; Vogt and Vogt, 1919; Nieuwenhuys et al., 2014). A recent paper by Glasser and Van Essen (2011) suggested utilizing MR imaging to partition the cortex based on myeloarchitecture (Geyer et al., 2011; Glasser and Van Essen, 2011; Glasser et al., 2016). It has been proposed that myelin and other compounds colocalized to myelin [such as lipids (e.g., cholesterol), free and myelin–bound water and iron] influence the longitudinal (T_1_) and transverse (T_2_ and T_2_^∗^) relaxation times (Koenig, 1991; Miot-Noirault et al., 1997; Schmierer et al., 2004; Callaghan et al., 2015).

The relative contribution of the individual compounds to the different MR parameters remains an active and important area of research (e.g., Rooney et al., 2007; Stuber et al., 2014; Callaghan et al., 2015). Water content (i.e., proton density), myelin (or more generally macromolecules) and iron have been identified as major determinants of T_1_ and T_2_^∗^ contrast across the brain and their distributions overlap significantly in many regions, especially in the cortex. The relative contributions of iron and myelin to the T_1_ and T_2_^∗^ parameters, however, differs; with myelin being the dominant contrast source in T_1_ (Rooney et al., 2007; Stuber et al., 2014; Callaghan et al., 2015) and iron in T_2_^∗^ maps (Fukunaga et al., 2010; Langkammer et al., 2010; Stuber et al., 2014). Stuber et al. (2014) report that iron has an average contribution of 10% to T_1_ (acquired at a field strength of 7 Tesla [7T]) in white matter and 36% in gray matter, while Callaghan et al. (2015) show that myelination is a better predictor of T_1_ variation than iron content. In contrast, the iron density strongly determines the T_2_^∗^ contrast not only in gray, but also in white matter in addition to the myelin concentration (Fukunaga et al., 2010; Stuber et al., 2014). Furthermore, the orientation of the myelinated fibers with respect to the magnetic field also influences the T_2_^∗^ contrast (Cohen-Adad et al., 2012).

Several studies showed that differences in MR intensity levels correlated well with observations in histological myelin-stained sections (Fatterpekar et al., 2002; Eickhoff et al., 2005; Bock et al., 2009; Geyer et al., 2011; Glasser et al., 2014; Stuber et al., 2014). By combining MRI with histology, these studies showed high concentrations of intra-cortical myelin in the primary areas, including the motor (M1), somatosensory (S1), visual (V1) and auditory (AC) cortices, but also in some higher order areas, such as MT^1^.

Several *in vivo* MRI mapping approaches were tested so far to study myeloarchitecture in humans, covering different magnetic fields (1.5T–7T) and MR parameters (T_1_-weighted [T_1_w], quantitative T_1_, T_2_^(∗)^-weighted^2^ [T_2_^(∗)^w], quantitative T_2_^(∗)^ and T_1_w/T_2_^(∗)^w). Cortical areas with higher myelination showed increased intensity levels in T_1_w (Bock et al., 2013) and quantitative R_1_ (=1/T_1_) images (Sigalovsky et al., 2006; Geyer et al., 2011; Dick et al., 2012; Sereno et al., 2013; Lutti et al., 2014) and reduced intensity in quantitative T_1_ (Tardif et al., 2015) and T_2_^∗^ images (Cohen-Adad et al., 2012). This implies that by computing the ratio of T_1_w and T_2_^(∗)^w image [i.e., T_1_w/T_2_^(∗)^w ratio], the contrast between the heavily myelinated areas and other regions can be enhanced (Glasser and Van Essen, 2011; De Martino et al., 2014). At the same time, this calculation eliminates the presence of receive bias fields.

When interpreting these myelin-related maps, it is important to recognize that T_1_w or T_2_^(∗)^w images are not quantitative markers of the underlying biochemical composition of the tissue, but are also influenced by the MRI sequence parameters, see for example Bock et al. (2013) and Lorio et al. (2016). MRI sequences utilized for weighted imaging might even mix different basic MRI parameters (Glasser et al., 2014). Moreover, the hardware setup, including the radiofrequency (RF) transmit and receive coils, influences not only the signal-to-noise ratio (SNR), but also the local image contrast. As a result, weighted images might incorporate contributions from transmit and receive RF fields in addition to proton density, relaxation rates, macromolecule concentrations and the MR parameter of interest. This may also hold for some quantitative approaches, which do not fully account for transmit inhomogeneities (e.g., see Marques et al., 2010). These issues are of particular importance when performing large-scale multi-site or longitudinal clinical studies that focus on, for example, quantitative differences in cortical myelin distribution reflecting microstructural changes due to experience, aging or disease (Draganski et al., 2011; Focke et al., 2011; Freund et al., 2013; Grydeland et al., 2013; Weiskopf et al., 2013; Callaghan et al., 2014; Droby et al., 2015).

These limitations can be tackled through a quantitative mapping approach by using, for example, the magnetization prepared 2 rapid acquisition gradient echoes (MP2RAGE) sequence (Marques et al., 2010). In addition, a multi-echo (ME) gradient-recalled echo imaging (GRE) sequence allows to obtain quantitative T_2_^∗^-maps by applying a mono-exponential fit (Cohen-Adad et al., 2012). The quantitative MRI maps are, however, also not necessarily free from effects unrelated to the underlying microstructural properties of the brain. For example, T_2_^∗^ maps are sensitive to non-local static field inhomogeneities near air-tissue interfaces, whereas T_1_ maps obtained with the MP2RAGE sequence (Marques et al., 2010) might be influenced by transmit field ($B_{1}^{+}$) inhomogeneities (Eggenschwiler et al., 2012). It is important to note that their weighted imaging counterparts have comparable field-inhomogeneity sensitivities, in addition to the ones mentioned earlier.

The different quantitative and non-quantitative MR parameters used so far for *in vivo* myelin mapping have not been systemically compared. The primary goal of the present study is, thus, to investigate the quantitative differences between several previously proposed MRI parameters (T_1_w, R_1_, T_2_^∗^w, T_2_^∗^, T_1_w/T_2_^∗^w, R_1_/T_2_^∗^) using 7T in terms of their variation across a group of healthy subjects and their reproducibility when a subject is scanned again. These are referred to as inter-subject coefficient of variation (COV) and intra-subject COV, respectively. The latter should ideally be low (i.e., low intra-subject myelination scan-rescan variability), while the former examines the effect of differential (inter-subject) myelination levels on each parameter and corresponding parcellability. Marques et al. (2010) showed high reproducibility of absolute T_1_ values acquired using the MP2RAGE sequence across multiple subjects and within one subject using different scanning parameters and scanners (e.g., 3T vs. 7T). Intra-subject COVs of T_1_ values obtained using MP2RAGE in deep-gray matter (dGM) regions ranged from 2.08% in the pulvinar to 2.89% in the substantia nigra (Okubo et al., 2016). The inter-subject reproducibility of cortical T_2_^∗^ maps acquired using a ME-GRE sequence was around 1.66% (Govindarajan et al., 2015), while a higher inter-site (applying the same sequence on the same subject at two different scanning sites) COV was observed for R_2_^∗^ (1/T_2_^∗^; 20.3%) and T_1_w (15.2%) maps. Lower inter-site COV was found for R_1_ (6%) maps. Note, that these inter-site COVs were based on lower-resolution (i.e., 1 mm^3^ voxel-size) R_1_, T_1_w and R_2_^∗^ maps acquired simultaneously (i.e., multi-parameter mapping, see Weiskopf et al., 2013) using 3T and therefore differ from the maps acquired using the MP2RAGE and/or ME-GRE sequences.

Compared to 3T, 7T allows the acquisition of sub-millimeter resolution data within a shorter time frame and without significant penalties to the SNR and contrast-to-noise ratio (CNR). In the currently applied scanning protocol, we aimed at as short as possible total scanning time while maintaining reasonable data quality at sub-millimeter resolution (0.7 mm × 0.7 mm × 0.7 mm) and evaluated SNR and CNR values and their spatial distribution for each parameter. Note that the T_1_w/T_2_w approach by Glasser and Van Essen (2011) is not included in the present investigation as acquiring whole-brain T_2_-weighted images at 7T is challenging due to inhomogeneous transmit profiles, power deposition limitations and long acquisition times. In addition, T_2_^∗^-weighted imaging has proven to be a comparable alternative for whole-brain high-resolution imaging of cortical myelination at 7T (Cohen-Adad et al., 2012; De Martino et al., 2014). However, T_2_ imaging at 7T may become more feasible as parallel transmission becomes routinely used.

In addition, the current study addresses several issues related to myelin-driven parcellation of the cortex using MR parameters. First, we quantify how reliably a specific vertex value can be parcellated based on its variation within subjects and the shape of the global distribution. Second, we compare – in the same subjects – the overall covariance between (weighted and quantitative) R_1_ (or T_1_w) and T_2_^∗^ and between both ratio images, along the cortical surface and at different cortical depths. Although we expect similar myelin-related patterns across all parameters as in previous studies, we hypothesize that, nevertheless, differences might exist since each MR parameter is potentially affected differently by the various microstructural properties, specific biases and noise. Second, as cortical parcellation requires the definition of borders between regions, we compare two widely used methods, based on threshold contours and surface gradients, to explore whether objective criteria for cortical parcellation can be derived or whether ambiguity is present and to what extent manual interaction is necessary.

## Materials and Methods

### Subjects and Data Acquisition

Ten healthy volunteers (age = 29.7 ± 6.3, between 24 and 42 years old, seven females) were included in this study. All subjects gave written informed consent in accordance with the Declaration of Helsinki. The protocol was approved by the Ethical Committee of the Faculty of Psychology and Neuroscience, University of Maastricht, the Netherlands. MR data were acquired using a whole-body 7T magnet (Siemens Medical Systems, Erlangen, Germany) and a 32-channel phased-array head coil (Nova Medical, Wilmington, DE, USA). High resolution (0.7 mm isotropic nominal voxel size) whole-brain quantitative T_1_ (T_1_) and T_1_-weighted (T_1_w) images were obtained with the 3D MP2RAGE sequence (Marques et al., 2010) with the following parameters: TR/TE = 5000/2.47 ms, TI_1_/TI_2_ = 900/2750 ms, α_1_/α_2_ = 5°/3° and generalized autocalibrating partially parallel acquisitions (GRAPPA) factor = 3 in the phase-encoding (PE) direction (anterior–posterior) with 24 references lines. Other acquisition parameters were: 240 sagittal slices, field of view (FOV) = 224 mm × 224 mm, matrix = 320 × 320 × 240, 6/8 partial Fourier in PE direction, non-selective RF excitation, readout bandwidth (BW) = 250 Hz/pixel, readout sample spacing = 6.9 ms and total acquisition time of 8:02 min. Whole-brain $B_{1}^{+}$ maps (2 mm isotropic nominal voxel size) were obtained using the saturation-prepared with 2 rapid gradient echoes (SA2RAGE) sequence (Eggenschwiler et al., 2012) with the following parameters: TR/TE = 2400/0.78 ms, TD_1_/TD_2_ = 580/1800 ms, α_1_/α_2_ = 4°/11° and GRAPPA factor = 2 in PE direction (anterior–posterior) with 24 references lines. Other acquisition parameters were: 88 sagittal slices, FOV = 256 mm × 256 mm, matrix = 128 × 128 × 96, 6/8 partial Fourier in PE direction, non-selective RF excitation, BW = 1300 Hz/pixel, readout sample spacing = 2.2 ms and total acquisition time of 2:16 min. Quantitative T_2_^∗^ (T_2_^∗^) and T_2_^∗^-weighted (T_2_^∗^w) images (0.7 mm isotropic nominal voxel size) were obtained from a multi-echo 3D GRE sequence with the following parameters: TR = 33 ms, TE_1_/TE_2_/TE_3_/TE_4_ = 2.53/7.03/12.55/20.35 ms, α_1_ = 11° and GRAPPA factor = 2 in PE direction (left-right) with 30 references lines. Other acquisition parameters were: 208 axial slices, FOV = 224 × 159, matrix = 320 × 227 × 208, 6/8 partial Fourier in phase and slice direction, slab-selective RF excitation, readout BWs = 290/210/170/90 Hz/pixel (different BWs were used to match SNR between the individual echoes), readout sample spacing = 4.5 ms and total acquisition time of 8:33 min. Dielectric pads containing a 25% suspension of barium titanate in deuterated water were placed proximal to the temporal lobe area to locally increase the transmit $B_{1}^{+}$ field and to improve its homogeneity across the brain (Teeuwisse et al., 2012). To analyze within-subject reproducibility for each parameter, data were acquired twice for a subset of the subjects (*N* = 3).

### Data Preparation

The T_2_^∗^ maps were obtained from the GRE data using a mono-exponential fit (f(TE) = S_0_e^-TE/T_2_∗^), whereas the T_2_^∗^w image was calculated by dividing the image at TE_4_ by the image acquired at TE_1_ to correct for receive bias fields. The T_1_w maps used in this study are MP2RAGE images calculated from the two image volumes (INV1 and INV2) acquired at TI_1_ and TI_2_ (see sequence details), which minimizes the effect of $B_{1}^{+}$ variations through space. A T_1_ map was calculated online by linear interpolation of the INV1 and INV2 images (see Marques et al., 2010, for more details). Both the T_1_w and T_1_ map were *post hoc* corrected for variations in $B_{1}^{+}$ using the same method as described in Marques and Gruetter (2013). All data were co-registered and resliced using a rigid-body transformation (six degrees of freedom) and 7th degree B-Spline interpolation to the T_1_w map to correct for subject motion between acquisitions using SPM 8 (Wellcome Department of Imaging Neuroscience, University College London, London, UK). Quantitative R_1_ and both ratio images (T_1_w/T_2_^∗^w and R_1_/T_2_^∗^) were generated using the mitools software package^3^. Note that we use R_1_ instead of T_1_ for comparison with the T_1_w images as the contrast is reversed between these two images (see Results for details).

### Processing Pipeline

**FIGURE 1:**
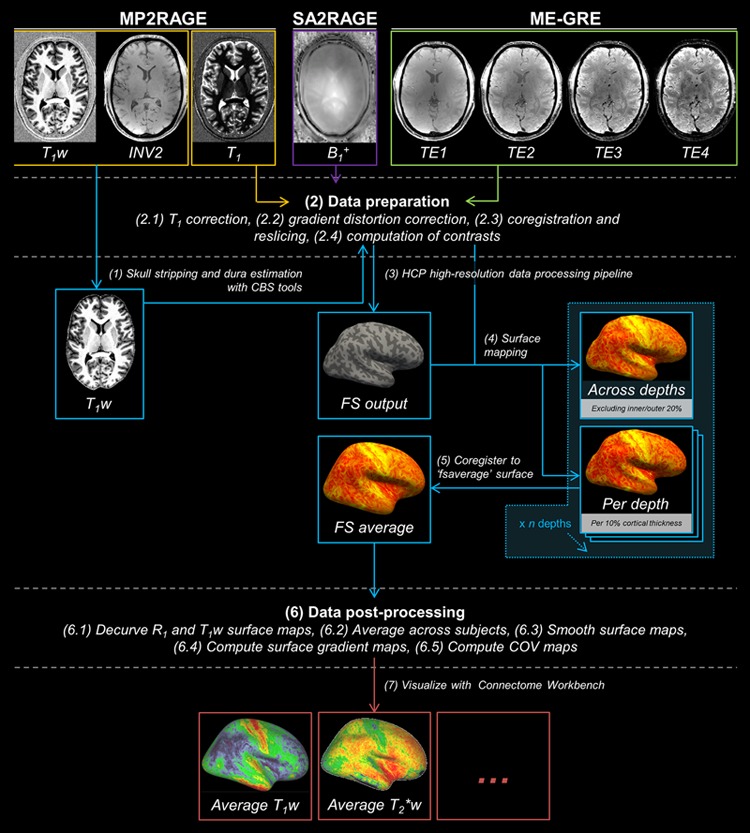
**The analysis pipeline for cortical myelin mapping using MP2RAGE (in orange), SA2RAGE (in purple) and ME-GRE (in green) data.** MP2RAGE images are used for reconstruction of the cortex (in blue). Skull stripping (1), data preparation (2), HCP high-resolution data processing pipeline (3), surface mapping and cortical depth sampling (4), coregistration to ‘fsaverage’ surface (5), data post-processing (6) and visualization (7) are performed in this order.

Several data pre-processing steps were performed to prepare MP2RAGE data for FreeSurfer 5.3.0-HCP^4^ processing (see **Figure 1** for illustration of the computational workflow). First, skull stripping was performed by obtaining a brain mask using the INV2 image. The INV2 image was used as it provides the best intra- and extra-cranial tissue contrast. Second, the generated brain mask was combined with a probability map of the dura mater for brain extraction. These initial steps were performed using MIPAV 7.1.1 (Center for Information Technology, NIH, Bethesda, MD, USA), JIST 3.0 (Johns Hopkins University, Baltimore, MD, USA) and CBS High-Res Brain Processing tools 3.0.5 (Max Planck Institute for Human Cognitive and Brain Sciences, Leipzig, Germany). Next, gradient distortion correction was applied on the brain extracted and other volumes using the gradient coefficients file provided by the scanner manufacture. This step is part of the Human Connectome Project (HCP) high-res analysis pipeline, which is especially designed to handle sub-millimeter resolution datasets (Glasser et al., 2013). The skull-stripped and gradient distortion corrected T_1_w volume was used as input to the HCP high-res analysis pipeline. As part of this pipeline, the T_1_w volume’s resolution was downsampled to 1 mm isotropic resolution and used for FreeSurfer’s recon-all method for cortical segmentation. Note, however, the final cortical surfaces were generated in the native resolution (0.7 mm isotropic). The resulting transformations matrices were also applied to the other parameter images for coregistration.

For each subject, all parameters were projected onto the surface using FreeSurfer’s mri_vol2surf function both (i) by averaging between 20 and 80% of the cortical thickness to reduce potential partial voluming with WM and CSF and, alternatively, (ii) by sampling at specific relative cortical depths between 0% (WM-GM border) and 100% (pial surface) of the cortical thickness with steps of 10%, resulting in 11 depths fractions. All surface maps were coregistered to the ‘fsaverage’ subject for further analyses. To avoid any curvature-related changes in the spatial distribution of the cortical T_1_w or R_1_ signal (Lutti et al., 2014), curvature-residualized maps of T_1_w and R_1_ variation were computed for each subject in MATLAB (R2013B, The MathWorks, Natick, MA, USA). Final surface maps were averaged across subjects (*N* = 10) for each parameter.

### Data Visualization

The surface maps were visualized using the Connectome Workbench v1.2.2 viewer (Washington University School of Medicine, Saint Louis, MO, USA) after conversion of the inflated surfaces and overlays to a compatible format. Non-cortical tissue in between hemispheres was masked using FreeSurfer’s parcellation scheme to avoid inappropriate scaling of the surface maps. A color map was chosen that optimally highlighted the contrast between lightly- and heavily myelinated areas, respectively.

### Regions of Interests

Several regions of interests (ROIs, see Supplementary Figure S1) were selected from the PALS-B12 atlas and FreeSurfer’s parcellation for a more detailed comparison between MR parameters and included heavily myelinated regions (probabilistic Brodmann areas [BAs] 1, 2, 3, 4, 17, 18, MT and transverse temporal gyrus [gTT] and sulcus [sTT]), moderately myelinated regions (BAs 6, 44, and 45) and a lightly myelinated region (BA29, i.e., retrosplenial cortex).

### Quantitative Data Analysis

Quantitative comparison between parameters was done for multiple parameters obtained using custom scripts written in MATLAB. First, the inter-subject (*N* = 10) and intra-subject (*N* = 3) coefficients of variation (COV) were computed using the vertex data from the averaged surface map. The inter-subject COV estimates the effects of myelination variability across subjects on each parameter, while the intra-subject COV assesses the scan-rescan reproducibility within subjects. In both cases, the COV was computed for each vertex.

(1) $${\text{COV}}_{\text{pv}}=\frac{\sigma_{\text{pv}}}{\mu_{\text{pv}}}\frac{1}{FWHM}$$

Inter-subject and intra-subject COVs (± inter-vertex standard error, i.e., SE) were calculated by dividing the standard deviation (*σ*) for each vertex (v) and parameter (p) by the mean value (*μ*) of that same vertex across subjects or time points, respectively. These values were than normalized to the full-width-half-maximum (FWHM), of the average cortical distribution (after rescaling the values between the 3rd and 97th percentiles to range between 0 and 1) for each parameter to take into account differences in the distribution of the values (see also the Discussion).

Second, we computed the parcellability variation (PV) for each vertex to determine how reliably a vertex value can be located within the cortical histograms and therefore how reliably it might be parcellated. The PV was calculated as follows:

(2) $${\text{PV}}_{\text{pv}}=\frac{\sigma_{\text{pv}}}{FWHM_{\text{p}}}$$

The PV was calculated by dividing the average (across subjects) scan-rescan standard deviation (*σ*) for each vertex (v) and parameter (p) by the FWHM of the average cortical distribution (before rescaling) and averaged for each region (± inter-vertex SE). As a result, a lower PV indicates a higher parcellability of the corresponding region.

Third, to determine the quality of the surface maps based on the contrast between heavily- and lightly myelinated regions, the CNR per unit of time was calculated vertex-wise for each region and parameter similar to Deistung et al. (2013):

(3) $${\text{CNR}}_{\text{pyv}}=\frac{\left|S_{\text{pyv}}-S_{\text{psv}}\right|}{\sigma_{\text{s+y}}}\frac{1}{\sqrt{t}}\;per\;unit\;time\;(\min).$$

CNR for each MRI parameter (p) was computed by dividing the absolute differences between the intensity of a vertex (v) in an ROI (y) and a randomly selected vertex value in a lightly myelinated (defined by its average value) neighboring region (s) by the inter-vertex SD of the signal intensities of all the pooled vertices from both regions (*σ*). The latter value served as an estimate of the noise. Different less myelinated regions proximal to the respective ROI were used to minimize the effects of possible residual transmit/receive bias fields on the observed inter-region contrast. Mean CNR (± inter-vertex SE) was calculated across all vertices for each parameter and region and converted to CNR per unit time, by normalizing it to the square root of *t* (minutes).

### Vertex-Wise Correlation between Parameters

The vertex-wise correlation (Pearson correlation *r*) between T_1_w and T_2_w, R_1_ and T_2_^∗^, R_1_/T_2_^∗^ and T_1_w/T_2_^∗^w was assessed to globally investigate their relationship. This was done on the data averaged across depths but also, separately, as a function of cortical depths. Vertices clearly affected by susceptibility differences due to B_0_-inhomogeinities and characterized by an average T_2_^∗^ lower than 0.024 s were excluded. For reference, average T_2_^∗^ value observed in the white matter of a single subject was approximately 0.027 s.

### Cortical Surface Pattern Analysis

Surface pattern analysis was performed to qualitatively compare between parameters. Here, we focused on the heavily myelinated regions close to the central sulcus and auditory cortex and compared the area size and location of these regions between the parameters, based on the group surface maps after pre-smoothing the data with a geodesic Gaussian kernel of 1 mm, using two approaches to define areal borders.

First, we compared the parameters based on threshold contours. Thresholds (%) were based on the area under the curve (AUC) of the global histogram between the 3rd and 97th percentile. However, as cortical myelin concentration is a continuous variable, there is no precise threshold value to determine cortical area borders. To determine the “subjectivity” (i.e., threshold dependency), we analyzed the area covered (% of vertices within specific ROIs) by quantifying the portion of vertices with intensity values equal to or higher than that defined by several thresholds, ranging from 60 to 90% of the AUC of the cortical histograms. Note, that, for this purpose only, we transformed the T_2_^∗^(w) data to R_2_^∗^(w), so that its profile across thresholds matches with those from the other parameters. Three thresholds (75, 80, and 85% of AUC) were eventually selected for visualization on the surface and quantification of overlap of areas between parameters (see Supplementary Material S1).

Alternatively, areas borders can be determined using the gradient of the parameters (Glasser and Van Essen, 2011; Glasser et al., 2016). For this, the pre-smoothed group surface maps were used to compute local maximum gradients using the ‘metric-gradients function’ within the Connectome Workbench command. In brief, at each vertex, the gradient is computed using a regression between the values of the vertex and of its neighboring vertices after spatially transforming them into a plane tangent. The gradient is then given by the slopes of the regression and reconstructed as a surface overlay.

## Results

### Comparing Myelin-Related Cortical Patterns Using Different Contrasts at 7T

**FIGURE 2:**
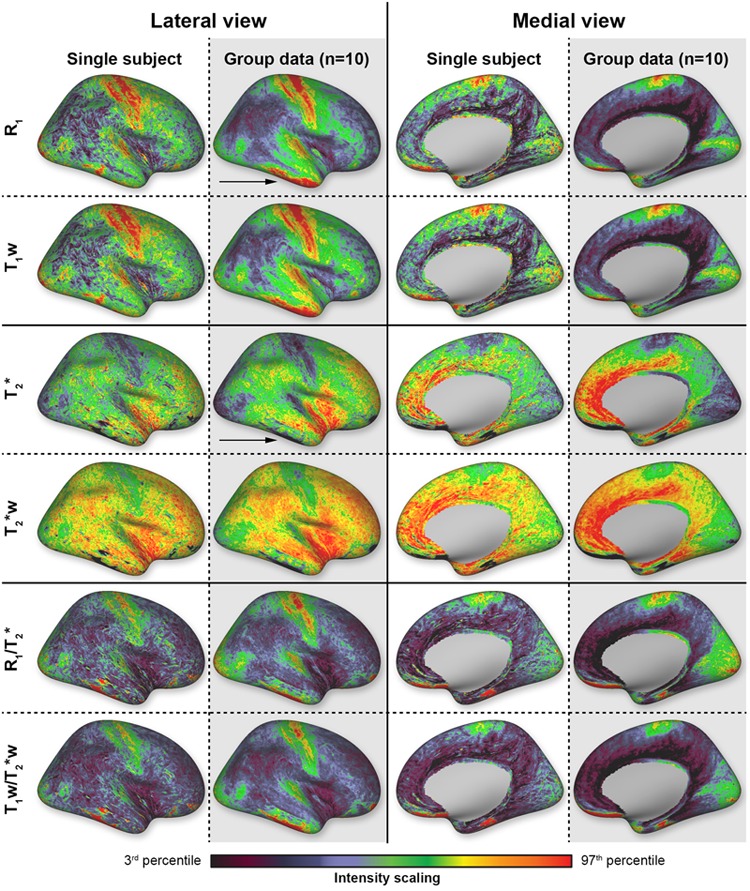
**Overview of the different parameters (rows) mapped on the reconstructed cortical surface.** Data are shown from both lateral and medial perspectives for a single-subject (white columns) and the average brain (*N* = 10, gray columns). Scaling is based on the 3rd and 97th percentiles.

In general, similar spatial myelin-related patterns are observed for all different parameters obtained (see **Figure 2** for the single-subject and group data). For simplicity, only the right hemispheres are shown as there were no evident differences between the left and right hemispheres. The values for the primary areas (including the somatosensory, motor, auditory and visual cortex) deviate most from the average values, which is in accordance with previous studies that showed strong myelination/increased cortical contrast in these areas at 1.5T, 3T, and 7T using different MR parameters (Sigalovsky et al., 2006; Glasser and Van Essen, 2011; Cohen-Adad et al., 2012; Bock et al., 2013; Sereno et al., 2013; De Martino et al., 2014; Lutti et al., 2014).

In all subjects (*N* = 10) and for all parameters, most dominant myelination-weighted contrast is observed along the posterior (S1, i.e., BA2 and BA3) and anterior (M1, i.e., BA4) cortices around the central sulcus (CS). This area extends from the paracentral lobule toward the lateral sulcus. Increased contrast levels are also observed beyond the lateral sulcus in the superior temporal gyrus and in particular Heschl’s gyrus (HG), encompassing the auditory cortex (AC). More posteriorly, toward the occipital lobe, increased myelination is also observed close to the middle temporal (MT) region. Other regions of the visual system located on the medial side are also characterized by increased contrast levels and cover almost entirely the medial occipital lobe (including V1 and V2). High levels of myelination are also observed in the posterior part of the cingulate cortex. Lower levels of myelination are seen in the frontal regions and the middle and inferior temporal gyri. This contrast, between areas with higher and lower myelination, is enhanced after calculating the ratio images for both the weighted and quantitative T_1_ and T_2_^∗^ images (lower two rows in **Figure 2**). Clear artifacts due to $B_{1}^{+}$ - and B_0_-inhomogeinities can be seen in the inferior temporal and frontal lobes (pointed out by black arrows in **Figure 2**) and these regions were, therefore, excluded from the quantitative analyses below.

### Inter- and Intra-Subject Variability

**FIGURE 3:**
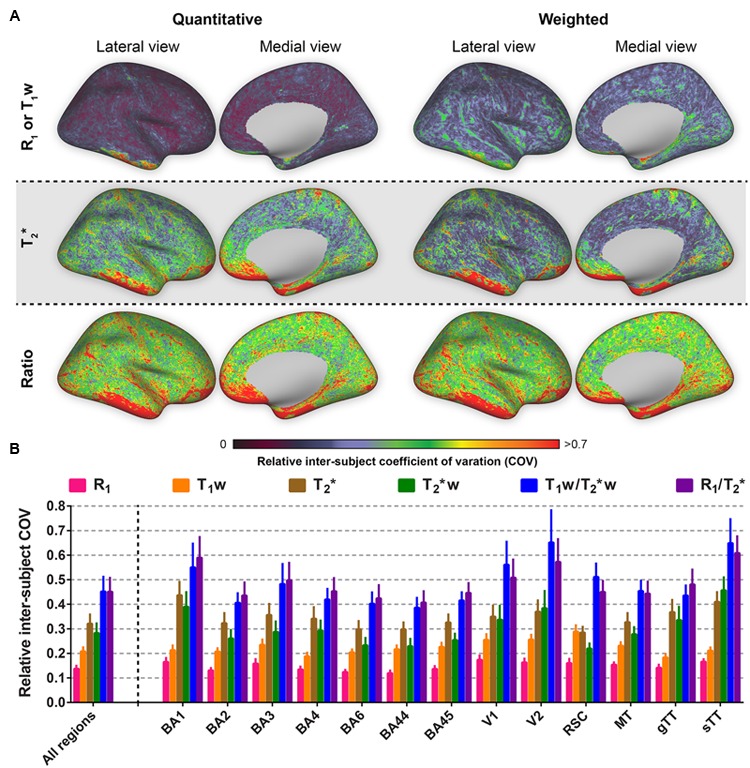
**Average relative inter-subject coefficients of variation (COV) comparison between the parameters investigated for the whole cortex, the selected regions of interest (ROIs) and all ROIs combined.** Relative inter-subject whole-brain COV maps are shown in **(A)**. Mean values (± inter-vertex SE) of inter-subject COV were extracted for each ROI (see Supplementary Figure S1) from the COV maps in **(A)** and plotted in **(B)**.

The relative inter-subjects (*N* = 10) and intra-subjects (*N* = 3) COV was determined to compare the different parameters in terms of similarity across healthy subjects and reproducibility within-subjects, respectively. **Figure 3A** shows the relative (i.e., normalized) inter-subject COV across the entire cortex. One can observe the lowest inter-subject COV for R_1_ (top row, left column) compared to the remaining parameters. Highest inter-subject COV can be seen for the ratio images (bottom row). The same trend is evident when analyzing the cortical ROIs individually (see **Figure 3B**): lowest COV for R_1_ (0.137 ± 0.012, averaged across regions) and highest for the quantitative T_2_^∗^ and weighted ratio images (0.451 ± 0.057 and 0.451 ± 0.060, respectively). A similar pattern is observed for the non-normalized COV values (Supplementary Figure S2). However, normalization especially corrects for the difference between T_2_^∗^ and T_2_^∗^w parameters (see **Table 1** for an overview of the FWHMs used for normalization).

**Table 1:** Overview of the FWHMs used to normalize the COV and to compute parcellability for each parameter.

Parameter	FWHM (after rescaling)	FWHM (before rescaling)
R_1_	0.4026	0.0503 s^-1^
T_2_^∗^	0.4788	0.0083 s
R_1_/T_2_^∗^	0.3888	4.4240 s^-2^
T_1_w	0.4573	339.0132 a.u.
T_2_^∗^w	0.3276	0.0764 a.u
T_1_w/T_2_^∗^w	0.3313	861.9166 a.u.

**FIGURE 4:**
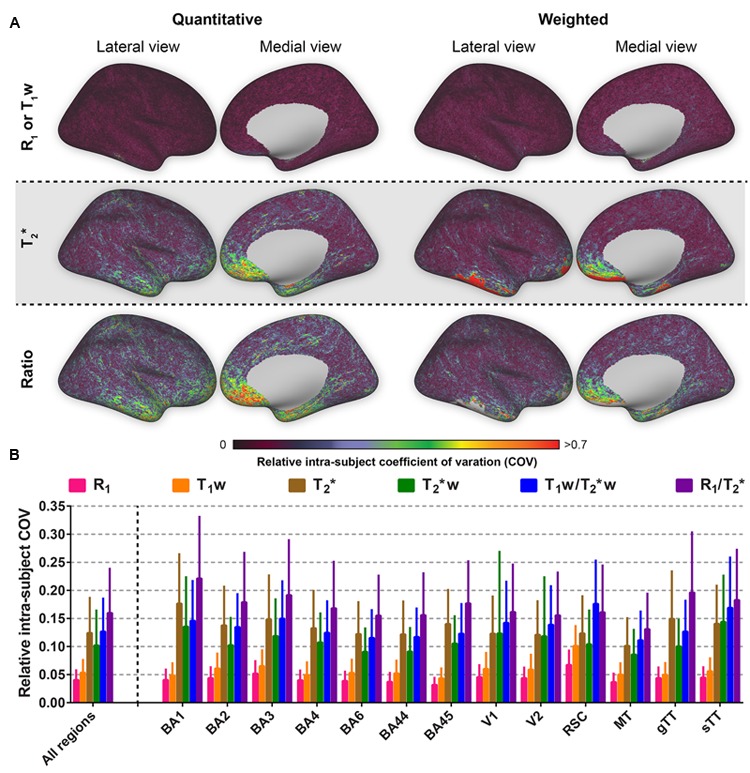
**Average relative intra-subject coefficients of variation (COV) comparison between the parameters investigated for the whole cortex, the selected regions of interest (ROIs) and all ROIs combined.** Relative intra-subject whole-brain COV maps are shown in **(A)**. Mean values (± inter-vertex SE) of intra-subject COV were extracted for each ROI (see Supplementary Figure S1) from the COV maps in **(A)** and plotted in **(B)**.

The intra-subject COV reveals that R_1_ is outperforming all other parameters in terms of reproducibility when comparing multiple images of the same modality acquired at different time points (see **Figure 4**). In addition, spatial intra-subject COV maps reveal lower variability for the T_1_w compared to the remaining parameters.

### Parcellability Variation

**FIGURE 5:**
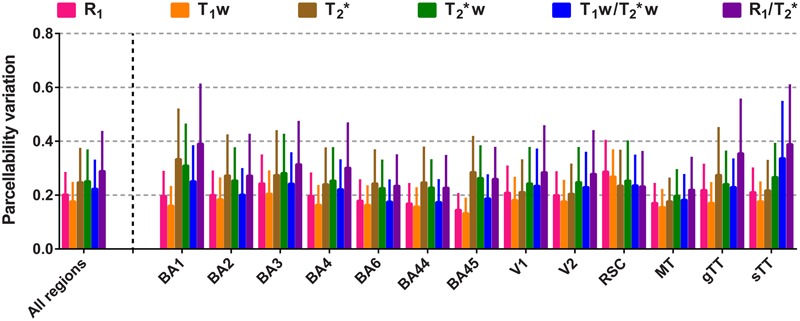
**Comparison of the parcellability variation (PV) between the parameters.** Mean PV values (± inter-vertex SE) are plotted for each parameter, region of interest (ROI) and all ROIs combined.

To compare the reliability of cortical parcellations based on the surface maps, the PV was computed for each parameter by dividing the vertex-specific intra-subject standard deviation (averaged across subjects, *N* = 3) by the FWHM of the corresponding global histogram (see **Table 1** for an overview of the FWHMs). Region-specific averages (± inter-vertex SE) and all regions together are plotted in **Figure 5**. In general (except for the RSC), R_1_ and T_1_w have the lowest (and comparable) PV, weighted against the other parameters. T_2_^∗^ and T_2_^∗^w are similar to each other, while highest scores were observed for the quantitative ratio.

### Contrast-to-Noise Comparison

**FIGURE 6:**
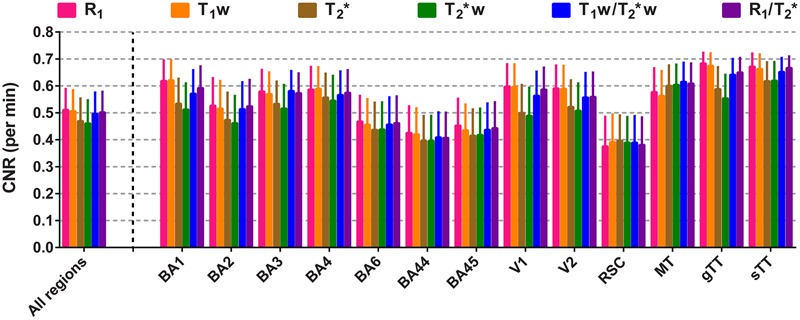
**Comparison of the mean contrast-to-noise ratio (CNR) per scanning time (in minutes).** Mean values of CNR per minute (± inter-vertex SE) are plotted for each parameter, region of interest (ROI) and all ROIs combined.

**Figure 6** shows the CNR per unit time (minute, mean ± intra-vertex SE) and per parameter for each of the different regions and all regions together. All parameters have comparable CNR per unit time. As for parcellability, the longitudinal relaxation constants (R_1_ and T_1_w) outperform the transversal ones (T_2_^∗^ and T_2_^∗^w) by having slightly higher CNR. When the ROIs are considered individually, CNR is lowest in the lightly myelinated regions BA6, BA44, BA45 and RSC and highest in the heavily myelinated regions like BA2, BA3, BA4, V1, V2, AC (sTT and gTT) and MT for all parameters. R_1_ and T_1_w show slightly higher CNRs for all heavy-myelinated regions, with the exception of MT where they score slightly lower.

### Vertex-Wise Correlation between Contrasts

**FIGURE 7:**
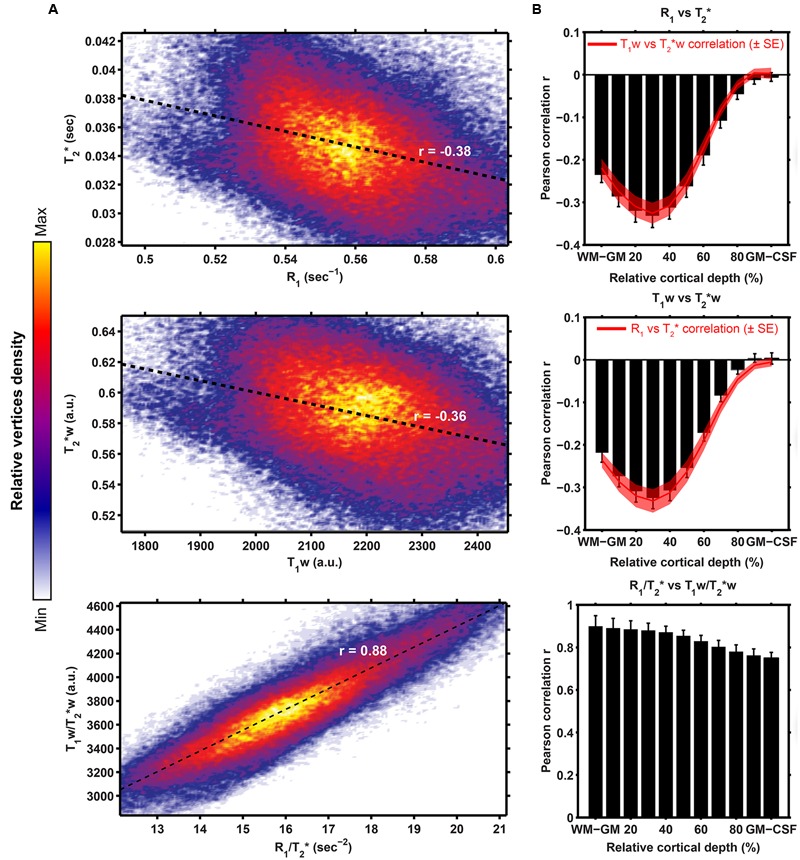
**Surface-based comparison of the correlations between R1 (or T_1_w) and T_2_^∗^ for both the quantitative (top row), weighted (middle row) as well as the ratio (bottom row) parameters.** For each pair of parameters, linear fits (black dashed line) were computed after plotting of the R_1_ (or T_1_w; *x*-axis in **A**) and T_2_^∗^ (*y*-axis in **A**) value of each vertex, where the color is determined by the relative vertices density. Pearson correlation *r* between both datasets was computed and plotted as a function of cortical depth (**B**, mean ± inter-subject SE).

**Figure 7A** shows the linear fits between the (i) R_1_ and T_2_^∗^ values (Pearson’s *r* = -0.38), (ii) T_1_w and T_2_^∗^w values (*r* = -0.36) and (iii) R_1_/T_2_^∗^ and T_1_w/T_2_^∗^w (*r* = 0.88), across all vertices. In all three cases, highest correlations are observed between the WM/GM border and mid-thickness level, but the correlation decreases strong when moving toward the GM/CSF border for R_1_ (or T_1_w) vs. T_2_^∗^ (or T_2_^∗^w, see **Figure 7B**).

### Threshold Dependency Analysis

**FIGURE 8:**
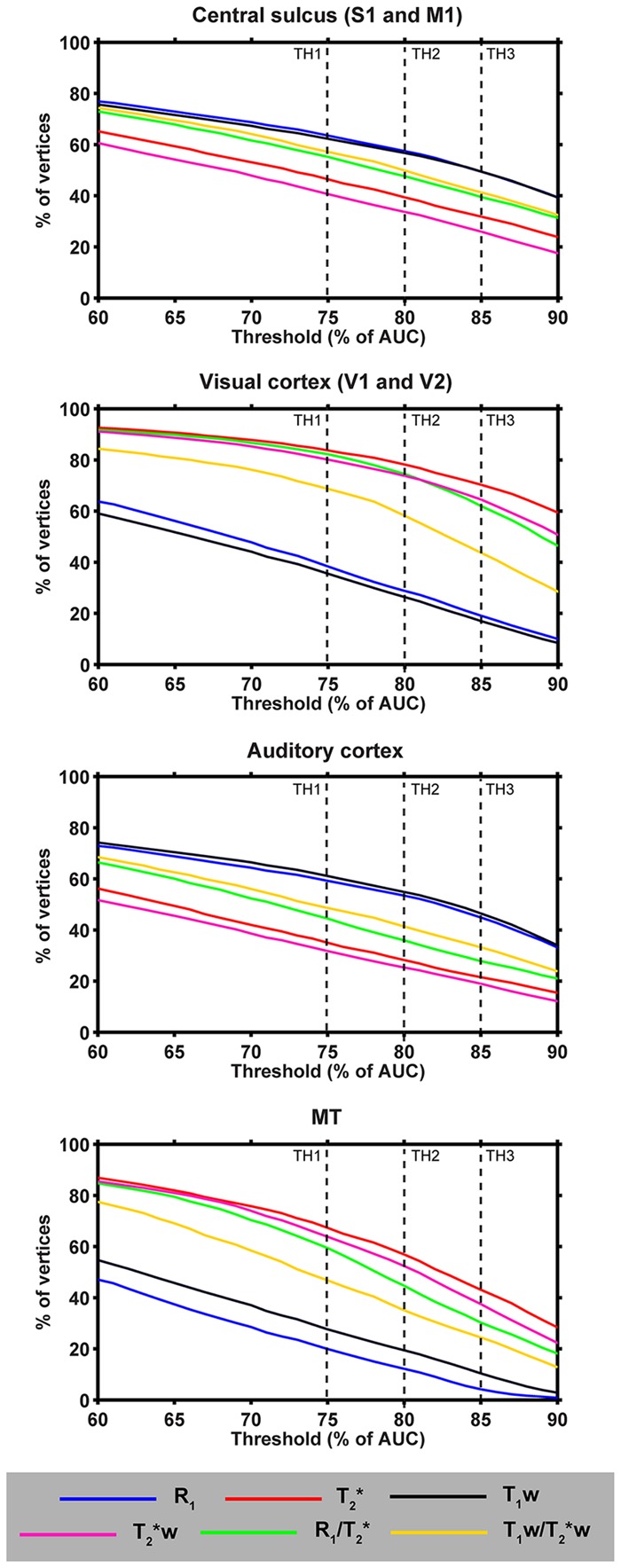
**Observer-independence analyses of the threshold contours approach.** For each parameter and different heavily myelinated regions (central sulcus [including M1 and S1], auditory cortex, visual cortex [including V1 and V2] and MT), the percentage of vertices (of the total region of interest) are plotted as a function of threshold level (60–90% based on the AUC of the corresponding histogram). Vertical dashed lines indicate the three thresholds used in **Figure 9**.

**Figure 8** displays the portion of vertices with intensity values equal to or higher than that defined by several thresholds (*x*-axes) for each parameter (colored solid lines) and strongly myelinated region. We focused on thresholds between 60 and 90% of AUC, as lower and higher thresholds are not relevant for our purpose since the transition between lightly- and heavy-myelinated regions lies within this range. The profiles revealed that T_1_w and R_1_ cover the largest surface areas with respect to CS and AC, independent of the threshold value, whereas T_2_^∗^(w) cover the smallest surface areas. In contrast, this pattern is opposite for the visual cortex and MT. Compared to R_1_ and T_1_w, a slightly larger variability of the slope within the visual cortex and MT was observed for the T_2_^∗^(w) and both ratio parameters. In general, the profiles of each parameter and region suggest that the differences between each parameter are relatively independent of the threshold.

### Surface Pattern Comparison

**FIGURE 9:**
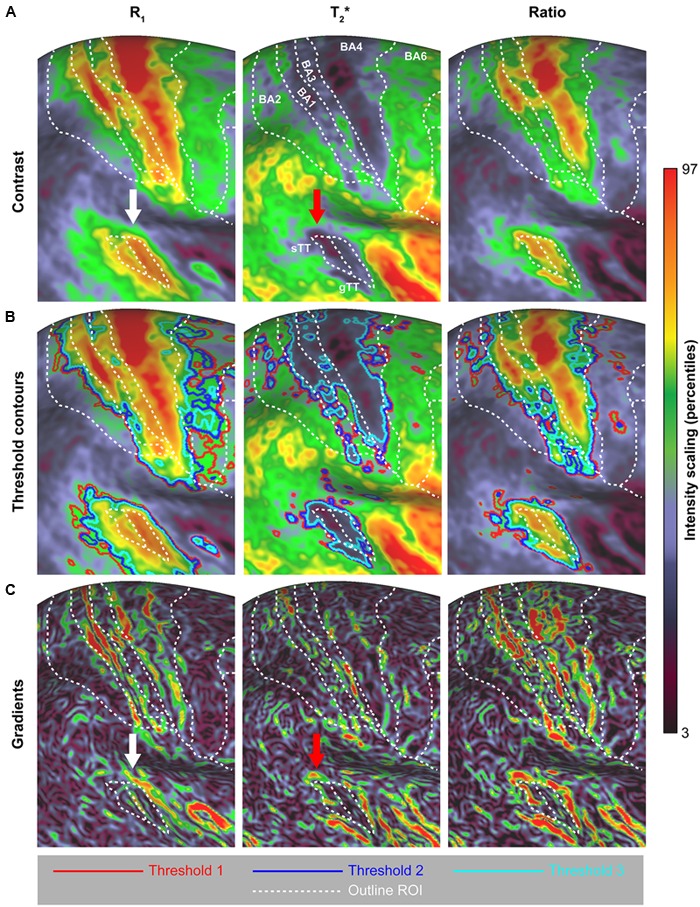
**Surface pattern comparison across R_1_, T_2_^∗^ and their ratio (A)**, based on threshold contours **(B)** and surface gradients **(C)**. To allow comparison between parameters, the boundaries of the regions of interest (ROIs) are superimposed. Supplementary Figure S4 shows the effect of applying different pre-smoothing kernel sizes on the group-average surface maps and subsequent gradient maps.

Here, we explored two approaches to delineate cortical areas based on their myelin content: using visual inspection of the location of (i) contours based on thresholds and (ii) strong intensity gradients on the surface relative to a specific delineation of regions. **Figure 8** shows a comparison between the quantitative parameters (see **Figure 9A**) using contours based on three threshold levels (red, blue and light-blue solid lines in **Figure 9B**). Based on visual inspection, the delineated areas around the CS and AC are largest for R_1_ and smallest for T_2_^∗^, independent of the threshold. These observations are confirmed by quantitative analysis as shown in the Supplementary Figure S3, where we focused on the total area of several regions (mm^2^) and differences between parameters (using the Jaccard coefficient as a metric to define overlap), including those in other regions (i.e., MT and visual cortex). Moreover, we observe a shift in the location of AC more posteriorly in the T_2_^∗^ parameter (see **Figure 8**, red arrow) compared to R_1_ (white arrow).

In addition to the threshold approach, surface maps were compared between different parameters by computing the local maximum gradients. The surface maps in **Figure 9C** emphasize the areal borders of different regions (especially BA1, BA4 and AC) based on the presence of strong intensity gradients across the surface for the different parameters. However, the gradient magnitude of these borders varies between parameters. For example, when comparing the magnitudes of the gradients among parameters, lower gradients are visible between BA4 and surrounding regions for T_2_^∗^. Clearer surface gradients are visible for T_2_^∗^ and especially the R_1_/T_2_^∗^ ratio when focusing on the posterior border of the AC. Overall, the borders that can be extracted from this data are not unambiguous and require user input (i.e., a choice of the gradient threshold value and filling-in gaps in the borders). Additional smoothing does not remove this ambiguity of the gradients and decreases the structural detail (see Supplementary Figure S4). Based on these gradient maps, no clear variation, however, is observed with respect to the spatial location of regions between parameters, as was observed for the threshold contours approach.

## Discussion

MRI has been used in the last three decades to visualize brain function, metabolites, and vasculature, white and gray matter anatomy. In particular, T_1_-contrast MRI has been utilized to segment cortical gray and white matter. Recently, it has been proposed that T_1_- and T_2_^(∗)^-weighted MRI can also be employed to map myelin not only in white matter but also within the cortex, which can be used to parcellate the brain and to delineate brain areas (Sigalovsky et al., 2006; Geyer et al., 2011; Glasser and Van Essen, 2011; Cohen-Adad et al., 2012; Dick et al., 2012; Bock et al., 2013; Sereno et al., 2013; De Martino et al., 2014; Lutti et al., 2014; Tardif et al., 2015; Glasser et al., 2016). However, no systematic comparison between the proposed approaches (i.e., T_1_-, T_2_^∗^-weighted images and their ratios as well as their corresponding quantitative MR parameters) has been performed so far. The goal of the current study is to fill this gap by evaluating the previously proposed *in vivo* myelin-mapping approaches. To achieve this, we acquired high-resolution multi-parameter imaging data (T_1_ and T_2_^∗^, weighted and quantitative) using 7T and performed cortical surface-based analyses to quantitatively compare the inter-subject variability, intra-subject reproducibility and CNR between parameters. In addition, we analyzed the correlation between T_1_ and T_2_^∗^ and their ratios and evaluated thresholding- and gradient contour-based approaches to delineate areal borders for cortical parcellation.

### Inter- and Intra-Subject Reproducibility

One important quantitative parameter to compare different parameters is the COV (i.e., the extent of variability in relation to the mean of the distribution), which can be used to evaluate the inter-subject variation and intra-subject reproducibility for each parameter. Since the basic biochemical parameters (such as myelin density) of tissue should not be significantly affected within the time frame of the different acquisitions, the intra-subject COV is indicative of non-biochemical variability in the image contrast. Note that unlike to the conventional method of calculating the COV (i.e., σ/μ), an additional normalization step was performed in the current study to take into account differences in the distribution of the values (within the 3rd and 97th percentiles) using the FWHM. For example, in the extreme case for which all absolute values of a parameter are projected into a single value in a weighted image, there is decreased sensitivity for myelin using the weighted approach. Thus, in comparing the reliability of quantitative and weighted MRI approaches (or different variants of weighted approaches), we propose that FWHM normalization of the distribution is performed for the COV comparison.

After normalization, the lowest inter- and intra-subject COVs are generally observed for R_1_, followed by T_1_w, T_2_^∗^w, T_2_^∗^ and are highest for both ratios. The non-normalized COVs, however, allow us to directly compare our results with those of earlier studies. One should bear in mind that these studies in some cases used different MR imaging approaches (e.g., FLASH images with specific magnetization preparations) with the sources of bias and variability that come with it. Nevertheless, the observed inter- and intra-subject R_1_ COV values within our GM regions (5.53 and 1.63%) are comparable with the inter-site COV (6%) observed earlier (Weiskopf et al., 2013) and the reproducibility (2.08%) observed for dGM structures (Okubo et al., 2016), respectively. Higher inter-site COV was also observed for the T_1_w (15.2%) vs. R_1_ (6%) parameter by Weiskopf et al. (2013). It should be noted that the T_1_w approach as presented here, is largely comparable to the quantitative R_1_ map in terms of acquisition strategy, i.e., they are based on the same data. Standard T_1_w (e.g., MPRAGE) images are much more likely to show effects from MRI acquisition characteristics (Bock et al., 2013; Lorio et al., 2016). In our case, the lower inter- and intra-subject COV for R_1_ might therefore be a consequence of the non-linear way that T_1_ values are computed from the T_1_w values in the MP2RAGE approach. In particular, a certain range of T_1_ values is translated into a broader range of T_1_w values, as illustrated in Figure 3 of the original paper by Marques et al. (2010), leading to a higher variation and lower reproducibility for the T_1_w maps compared to the R_1_ maps. This, together with the fact that R_1_ changes with age (Draganski et al., 2011; Grydeland et al., 2013; Callaghan et al., 2014) may explain the smaller difference observed between the R_1_ and T_1_w intra-subject COV maps compared to the inter-subject COV maps, considering the age range of our study population (24–42 years). The highest COV for both ratio images could be explained by the fact that noise from both R_1_ (or T_1_w) and T_2_^∗^ measures propagate into the corresponding ratio images, leading to increased variability among subjects. Noise may be especially present in the T_2_^∗^ maps, as noise enhancement due to intensity fluctuations (i.e., increased SD) across echoes when fitting the GRE data possibly caused the higher (non-normalized) COV compared to the T_2_^∗^w maps and previous reported values based on data acquired similarly (Govindarajan et al., 2015). However, Govindarajan et al. (2015) used a 2D GRE sequence with anisotropic voxels and a high in-plane resolution (0.3 mm × 0.3 mm × 1 mm), therefore allowing a longer TR (2020 ms) to acquire more TEs (*N* = 12), while boosting the SNR. Acquisition of GRE data using an increased number of echo times to enhance the T_2_^∗^ fitting procedure significantly improves the COV. However, this also considerably increases the scanning time, which we kept as short and similar to the MP2RAGE as possible. For instance, in the Govindarajan et al. (2015) study covering only part of the brain, the GRE acquisition time was more than double of ours.

Note that for both quantitative R_1_ (or T_1_w) and T_2_^∗^, the spatial inter-subject COV maps are ideally not influenced by RF transmit field inhomogeneities. In case of R_1_ (and T_1_w) measures, possible deviations in the $B_{1}^{+}$ across subjects are removed by *post hoc* correcting the MP2RAGE data using the acquired $B_{1}^{+}$ -map (Eggenschwiler et al., 2012; Marques and Gruetter, 2013). For the ME-GRE data, the $B_{1}^{+}$ -inhomogeneities are removed from the quantitative T_2_^∗^ image by the virtue of the fitting process that separates all non-T_2_^∗^ contributions into a separate component. The division of ME-GRE images acquired at different echo times and the fact that the same flip angle was used for all readouts ensures that the resulting T_2_^∗^w image is also free from RF bias fields. Nevertheless, for the T_2_^∗^(w) COV maps, higher values are especially observed in more confined regions that are potentially affected by an increased density of veins (e.g., V1/2) or large arteries (e.g., RSC and AC). As a result, voxels in these regions may be influenced by strong artifacts, such as susceptibility variations and increased partial voluming, leading to significantly larger variations in the ME-GRE data within and across subjects close to the GM/CSF border. Also, the well-known B_0_-orientation dependence of the T_2_^∗^ signal could have induced a slight decrease in the reproducibility of the T_2_^∗^(w) maps (Cohen-Adad et al., 2012).

### Parcellability Variation

The FWHM of the distribution is a marker for the sensitivity of an MR parameter to differences in cortical values, on its own, if the image is not dominated by noise. However, in order to estimate how reliably a vertex value can be located within the cortical histograms and therefore how reliably it might be parcellated, a measure of noise should be taken into account. For example, the average intra-subject standard deviation for that specific vertex can serve as an estimate for non-biochemical variability in the image contrast. In the current study, a lower PV (i.e., a small standard deviation for a large FWHM) indicates a higher reliability to isolate that vertex’s value. The longitudinal relaxation constants (R_1_ and T_1_w) perform similarly better than the transversal relaxation constants (T_2_^∗^ and T_2_^∗^w). The observed difference is predominantly induced by the lower reproducibility of the T_2_^∗^ and T_2_^∗^w maps, since an advantageous (rescaled) FWHM was observed for the cortical T_2_^∗^ distribution. This interpretation can also be extended to both ratio images. Taken together, the present data suggests that R_1_ and T_1_w maps are more reliable in parcellating the cortex.

### Contrast-to-Noise Ratio

For the definition employed in the current paper, CNR provides an additional indication of the sensitivity of each parameter to differentiate between heavily- and lightly myelinated ROIs based on their average value and noise. Highest CNRs were observed for heavily myelinated regions (e.g., BA1, visual cortex and auditory cortex) and lowest for lightly myelinated regions (e.g., BA44, BA45 and RSC). Although, different MP2RAGE parameters (e.g., TR, TI_1_ and flip angles) were used than previously proposed for optimal CNR (Marques and Gruetter, 2013) to decrease the scan duration, R_1_ and T_1_w maps were characterized by the highest CNR across most regions. Lowest CNRs were observed for both the T_2_^∗^ and T_2_^∗^w parameters, which are not solely explained by differences in inter-subject variability, considering the fact that the highest variation was observed for both ratio images. However, this difference in CNR between the longitudinal (R_1_ and T_1_w) and transversal [T_2_^∗^(w)] parameters may be caused by differences in their biochemical basis, with myelin and iron being the dominant contributors to the cortical differences in R_1_ and T_2_^∗^, respectively (Stuber et al., 2014). This may also explain the smaller regional differences between R_1_ (or T_1_w) and T_2_^∗^(w) CNR in the lightly myelinated regions. In the RSC, lower CNR is observed for R_1_ and T_1_w, which is in agreement with the fact that previous studies reported highest myelin content in its layers close to the pial surface, which was excluded in our surface analysis, and lowest myelin content in its middle layers (Fatterpekar et al., 2002). However, since the CNR involves the absolute differences between two regions, artifacts induced by a large vein (*vena magna cerebri*) lead to significantly lower T_2_^∗^ values in the RSC, and may account for an artificial enhancement of CNR for the T_2_^∗^(w) maps in this region.

### Whole-Cortex Myelin-Related Pattern

The current data demonstrates good agreement in terms of the observed general myelin-related pattern across all investigated MR parameters and with earlier studies. As in previous studies, the primary areas, including M1, S1, V1 and AC, differed in their T_1_- or T_2_^∗^-values from the average values throughout the cortex. Noteworthy is the shift more posteriorly (i.e., corresponding to the gyral wall of Heschl’s gyrus) that we observed with respect to the localization of the auditory core (here defined as the part of AC with the highest degree of myelination) in the T_2_^∗^ compared to T_1_ parameter. This difference was present in both the single-subject and group data and can therefore not be explained solely by misalignment due to the averaging. B_0_-orientation dependence of T_2_^∗^ signal (Cohen-Adad et al., 2012) may have induced the slight difference in locations, but the precise effects of B_0_-orientation remain to be established across the entire cortex. R_1_ (or T_1_w) and T_2_^∗^(w) are especially (negatively) correlated in the deeper layers, which are generally characterized by the highest degree of myelination (Ding et al., 2016). Differences in the myelination across layers between the gyral wall and gyral crown of the Heschl’s gyrus may possibly explain the observed difference in location of the auditory core between R_1_ and T_2_^∗^.

The presented maps can be used to parcellate the whole brain into distinct regions based on strong changes in myeloarchitecture across the cortex (Geyer et al., 2011; Glasser and Van Essen, 2011; Glasser et al., 2016). However, since MR parameters values are by nature continuous, defining areal borders between regions with a distinct myeloarchitecture (i.e., characterized by a strong change in an MR value) requires an additional criterion to make the continuous value distribution to discontinuous border delineation. Two methods to define transitions between cortical regions were explored based on either (i) the contours that track vertices characterized by specific values (thresholds) or (ii) borders that track the peak locations of strong surface-based local gradients. Whereas the latter approach is argued to be observer-independent (Glasser et al., 2016), the first approach allows comparative analyses of the surface patterns between parameters as it provides continuous contours, but may also be more affected by the chosen threshold. Although the observer-dependence of the proposed threshold approach was minimized as much as possible, confounding factors like local artifacts, outliers or global differences in the shape of the histograms may lead to misleading conclusions. Nevertheless, our analysis showed that any choice of value between the 60th and 90th percentage of the AUC will not dramatically influence the final conclusions of surface pattern analyses as done in this paper and may therefore be adjusted according to the characteristics of the ROI. Interestingly, the differences between parameters (e.g., R_1_ vs. T_2_^∗^) in the surface area delineated by these thresholds, were not uniform across regions. This may possibly be induced by differences in their biochemical composition (e.g., higher tissue iron content) or due to a higher density of blood vessels (leading to lower T_2_^∗^(w) values) between the visual areas and somatosensory cortex, for example, respectively.

Alternatively, areal borders defined by the peak locations of myelin content change across the surface may provide additional supporting and more fine-scale information, such as the border between BA3 and BA4 or of the auditory core. However, as we demonstrate in the current work, the gradient method itself suffers from shortcomings and does not provide a completely objective approach for border definitions. The observed borders vary in quality depending on the parameter and are not continuous, therefore demanding observer input when comparing parameters (i.e., a choice of the gradient threshold value and filling-in gaps in the borders). This approach seems to be especially appropriate in group studies after including a significantly higher amount of subjects as demonstrated by Glasser and Van Essen (2011) and Glasser et al. (2016). Moreover, subsequent inter-subject surface registration methods that use multiple modalities (e.g., T_1_, thickness and curvature) will additionally improve the quality of the gradients (Robinson et al., 2014; Tardif et al., 2015). Studies that want to localize and/or characterize the structural extent of specific regions within single-subjects (e.g., to restrain their functional analyses) would, however, not substantially benefit from this method as compared to the threshold method based on the current data. Within a single subject, more continuous gradients can be obtained by applying a higher smoothing factor across the surface, however, at the expense of sacrificing structural detail. In summary, given the continuous distribution of MRI values, both the threshold and the gradient methods require user input to define areal borders, and more improved algorithms and comparison with histology are necessary for objective, observer-independent cortical parcellation at the single-subject level.

### Technical Considerations

Despite the improvements in post-processing software tools and pipelines, accurate delineation of the gray and white matter borders is still difficult and manual correction of segmentation errors may have to be performed. Automatic segmentation of MP2RAGE data acquired at 7T is especially challenging in the lower parts of the brain that suffer from a low $B_{1}^{+}$ -field and susceptibility effects. In the current study, these effects were partially eliminated by using dielectric pads (O’Brien et al., 2014) and by applying corrections for $B_{1}^{+}$ inhomogeneities (Eggenschwiler et al., 2012). Erroneous segmentation may lead to imperfect mapping of the parameters in these areas (e.g., at variable cortical depths) and misinterpretation of the data (Lutti et al., 2014; Waehnert et al., 2015).

## Summary

Our data acquired using a time-efficient imaging protocol (<20 min) strengthens the robustness of myelin-related cortical mapping across different (i) magnetic field strengths, (ii) MR parameters and (iii) sequences. Despite the similarities that were observed between R_1_ (or T_1_w) and T_2_^∗^ quantitative and weighted MRI with respect to myelination patterns and CNR per unit time, most importantly and optimally, MR parameters should be characterized by a high intra-subject reproducibility with minimal contamination by non-biochemical factors (e.g., MRI acquisition-related variations) and high reliability for cortical parcellation (i.e., low PV). Here, R_1_ was characterized by (i) a low COV both within- and between-subjects and (ii) low PV, making it more suitable as a myelin-related MR biomarker compared to the other tested MR parameters for studies in learning, gender, age and diseases effects on the brain’s microstructure. However, the most important argument to take into account when choosing sequences is that quantitative imaging (such as R_1_, T_2_^∗^ or their ratio) allows detecting absolute differences within and between subjects. Detecting heavily- and lightly myelinated areas using weighted imaging relies on the fact that these areas differ in their MRI intensity from the average values in the brain. That is, the normalization of myelination distribution based on weighted-MRI intensity is typically done within a subject. However, it is conceivable that subjects differ not only in their relative distribution, but also in their absolute myelination, for example, due to disease and, in these cases, quantitative MRI parameters are most likely more suitable for inter-subject comparison.

## Author Contributions

RH, DI, and KU designed the study. RH and DI acquired the data. RH performed the analyses and wrote the paper. DI, EF, and KU contributed to writing the paper.

## Conflict of Interest Statement

The authors declare that the research was conducted in the absence of any commercial or financial relationships that could be construed as a potential conflict of interest.

## Footnotes

1 Note that although myelin is not the only contributor to the MRI parameters investigated, following the current terminology, we associate the cortical distribution of T_1_ and T_2_^∗^ values with myelin patterns.

2 In the following, T_2_^(∗)^ represents both T_2_ and T_2_^∗^ and the weighted approaches are denoted by the index “w.”

3 http://od1n.sourceforge.net/

4 http://surfer.nmr.mgh.harvard.edu/
