# Supplementary material for: Reproducibility and Reliability of Quantitative and Weighted T1 and T2∗ Mapping for Myelin-Based Cortical Parcellation at 7 Tesla
Source: Front Neuroanat. 2016 Nov 18;10:112. doi: 10.3389/fnana.2016.00112 (PMC5114304; doi:10.3389/fnana.2016.00112)
Supplement: Supplementary file 1 [file Data_Sheet_1.PDF]

Supplementary Material

Reproducibility and reliability of quantitative and weighted T_1 and T_2* mapping for myelin-based cortical parcellation at 7 Tesla

Roy A.M. Haast[1], Dimo Ivanov[1], Elia Formisano[1] and Kâmil Uludağ[1*]

*** Correspondence:** Kâmil Uludağ (kamil.uludag@maastrichtuniversity.nl)

1 Supplementary Data

1.1 "Subjectivity" analysis

1.1.1 Materials and methods

For each inter- (e.g. R_1 vs. T_2*) and intra- (e.g. R_1 vs T_1w) comparison, threshold and ROI, we (i) quantified the number of vertices and consecutively the total surface area (mm^2) using MATLAB; (ii) obtained the total area covered by the union of both parameters and (iii) retrieved the overlap percentage between parameters based on the number of vertices within each corresponding outline. This metric is also known as the Jaccard similarity coefficient (J) and can be expressed using:

$$J_{y,T_{A,B}}(A,B) = \frac{\left|A_{y,T_A} \cap B_{y,T_B}\right|}{\left|A_{y,T_A} \cup B_{y,T_B}\right|} \quad (1)$$

Here, the total area for each ROI (y) where parameters A and B (defined using threshold T_A or T_B, respectively) intersect was divided by the total area for the union of A and B. Subsequently, the total surface area and percentage of spatial overlap were then averaged across the three different thresholds to reduce the effect of noise on the cortical distribution of each parameter (and subsequent interpretations).

1.1.2 Results

Total surface area (cm^2) for each of the regions and thresholds were subsequently plotted per parameter in Supplementary Figure 3B and the computed spatial overlaps (%, averaged across thresholds) between these areas are shown in Supplementary Figure 3C. Overall, higher spatial overlaps were observed between the weighted and quantitative images of the same parameter (i.e. intracontrast comparison) compared to the overlap between images from different parameters (i.e. intercontrast comparison).

2 Supplementary Figures

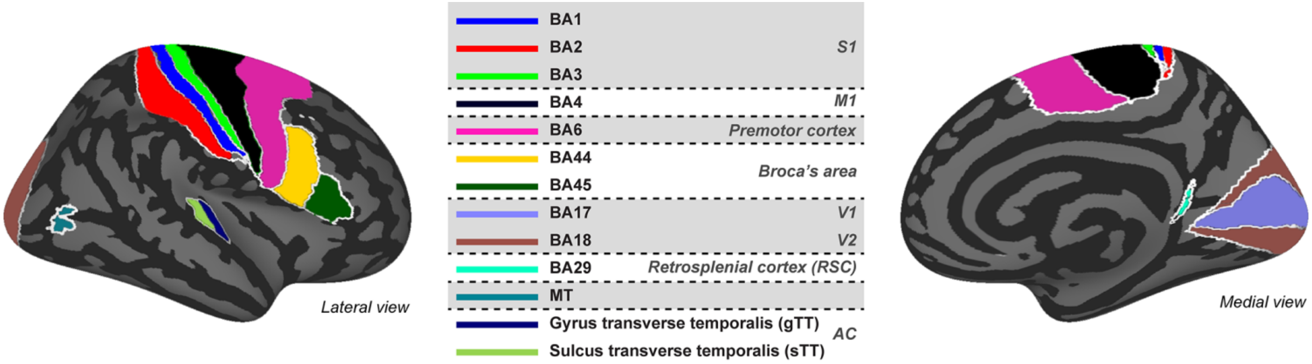

Supplementary Figure 1. Overview of the different regions of interest (ROIs) used for analyses.

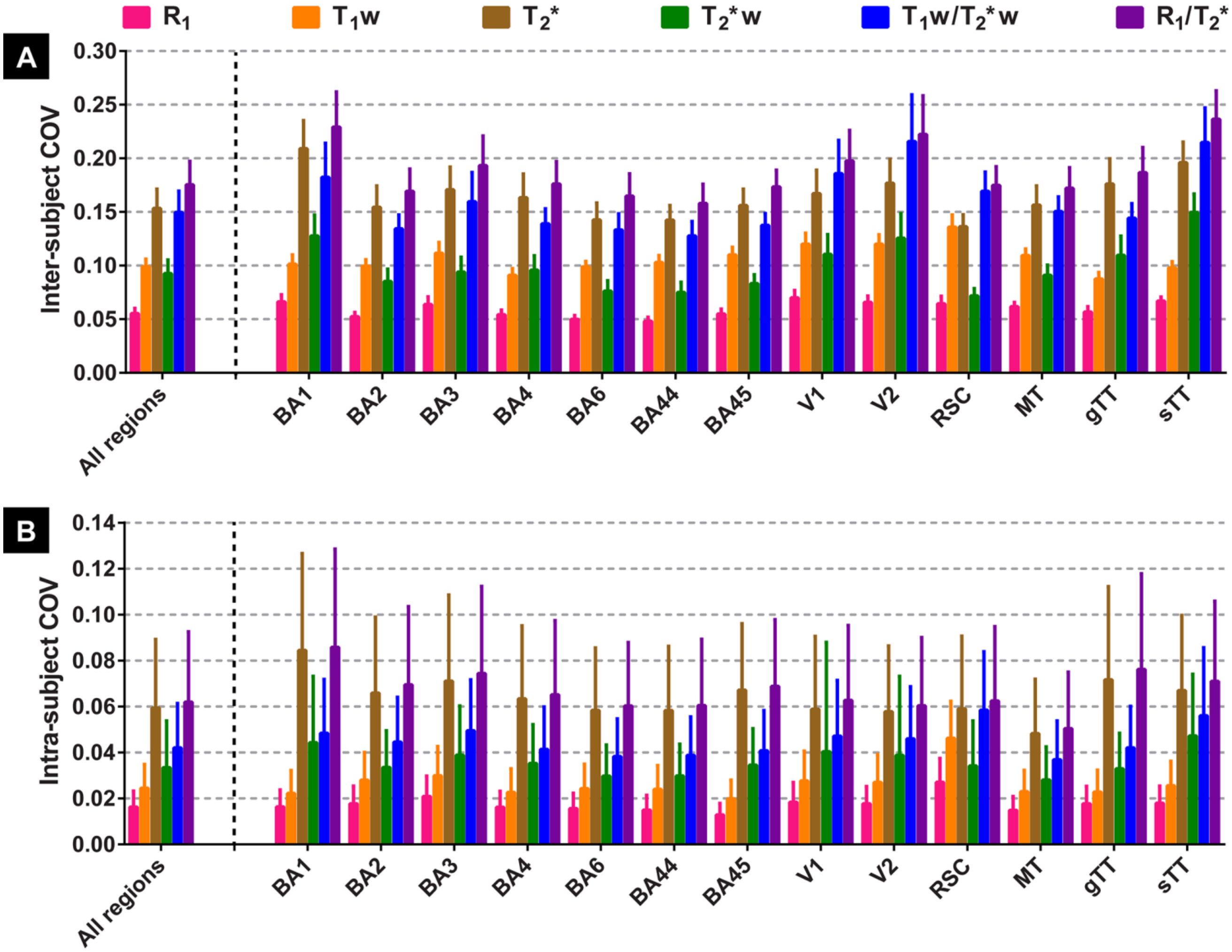

Supplementary Figure 2. Average, non-normalized, inter- and intra-subject coefficients of variation (COV, A and B, respectively) comparison between the parameters investigated for the selected regions of interest (ROIs) and all ROIs combined.

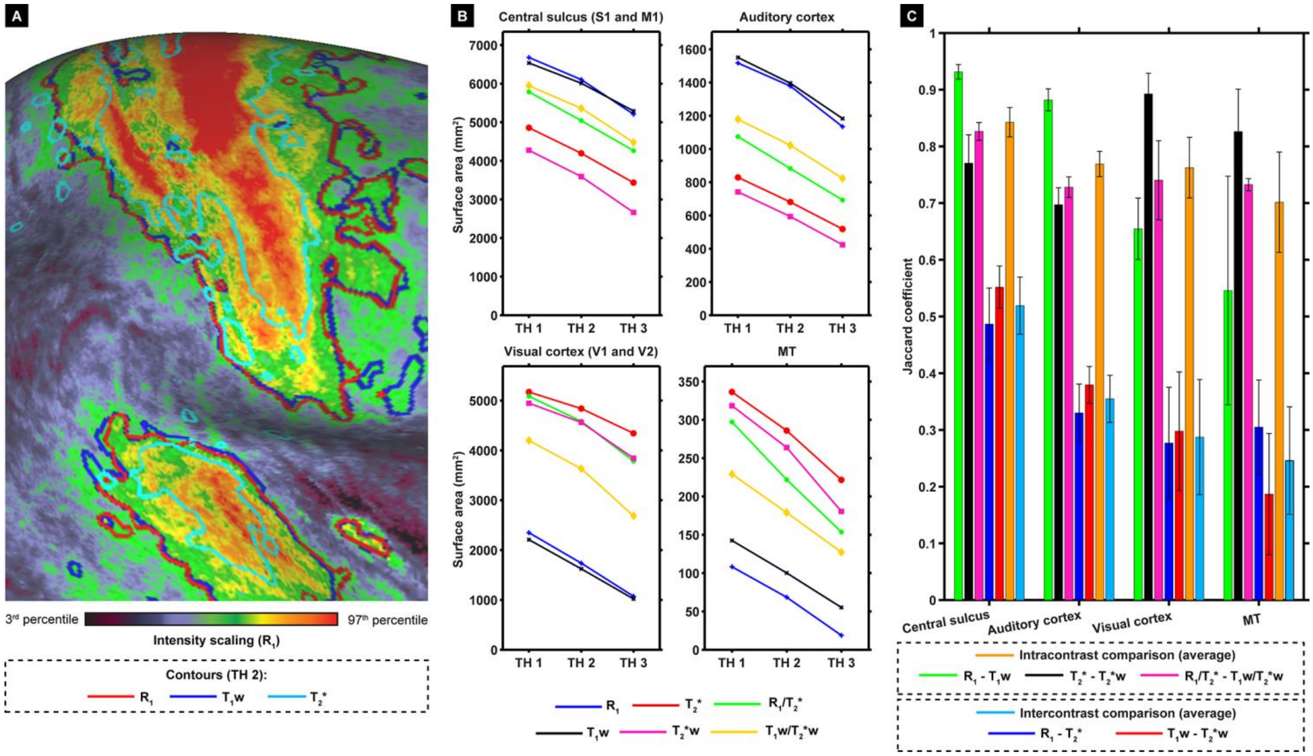

Supplementary Figure 3. Surface pattern comparison based on the threshold contours. For example, panel A shows the comparison within the central sulcus (including M1 and S1) and auditory cortex between R_1, T_1w and T_2* surface maps, based on their 2nd threshold contours (red, blue and light blue solid lines, respectively) overlaid on the R_1 surface map. The total surface area within the threshold was computed for each region (see B). The percentage where the areas within both contours were overlapping, with respect to the total area of both parameters combined, was plotted in C for each comparison and region. In addition, the overlap between parameters were combined to obtain intercontrast (T_1w vs T_2*w and R_1 vs. T_2*) and intracontrast (T_1w vs. R_1, T_2*w vs. T_2* and T_1w/$_T$2*w vs. R_1/T_2*) averages.

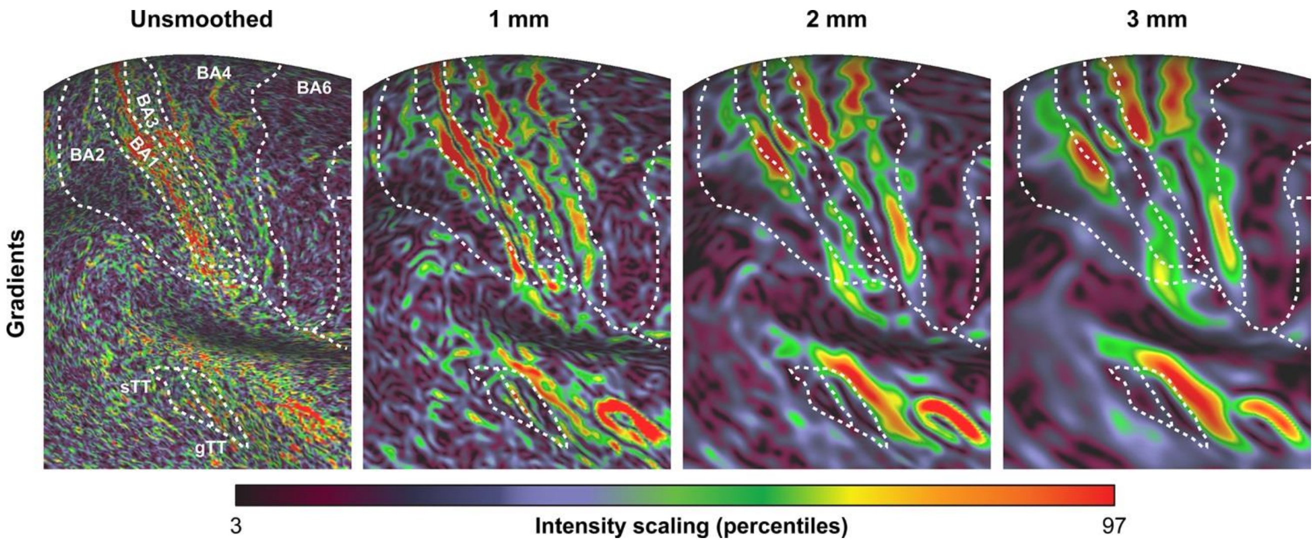

Supplementary Figure 4. Surface pattern comparison based on gradient maps from the R_1 parameter after applying different smoothing levels. Local maximum gradients maps were computed based on the group average R_1 surface map (see Figure 2) after applying different smoothing kernels: unsmoothed, 1 mm, 2 mm or 3 mm. No significant improvement above a kernel size of 1 mm can be observed.
