# Supplementary figures and images for: Reproducibility and Reliability of Quantitative and Weighted T1 and T2∗ Mapping for Myelin-Based Cortical Parcellation at 7 Tesla
Source: Front Neuroanat. 2016 Nov 18;10:112. doi: 10.3389/fnana.2016.00112 (PMC5114304; doi:10.3389/fnana.2016.00112)

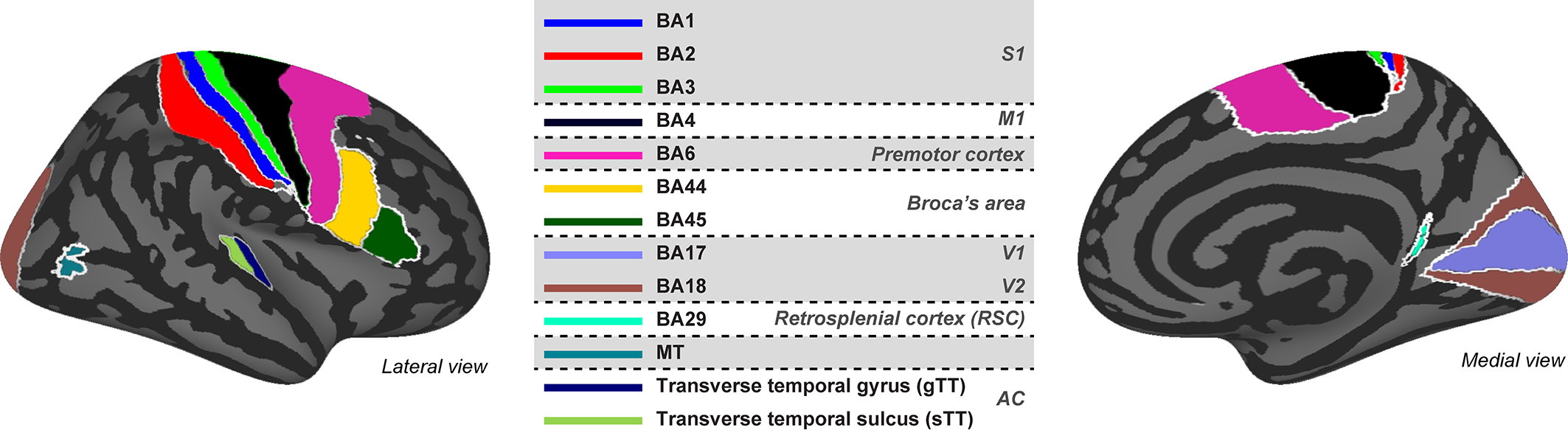

Supplement: Supplementary file 2 [file Image_1.TIF]

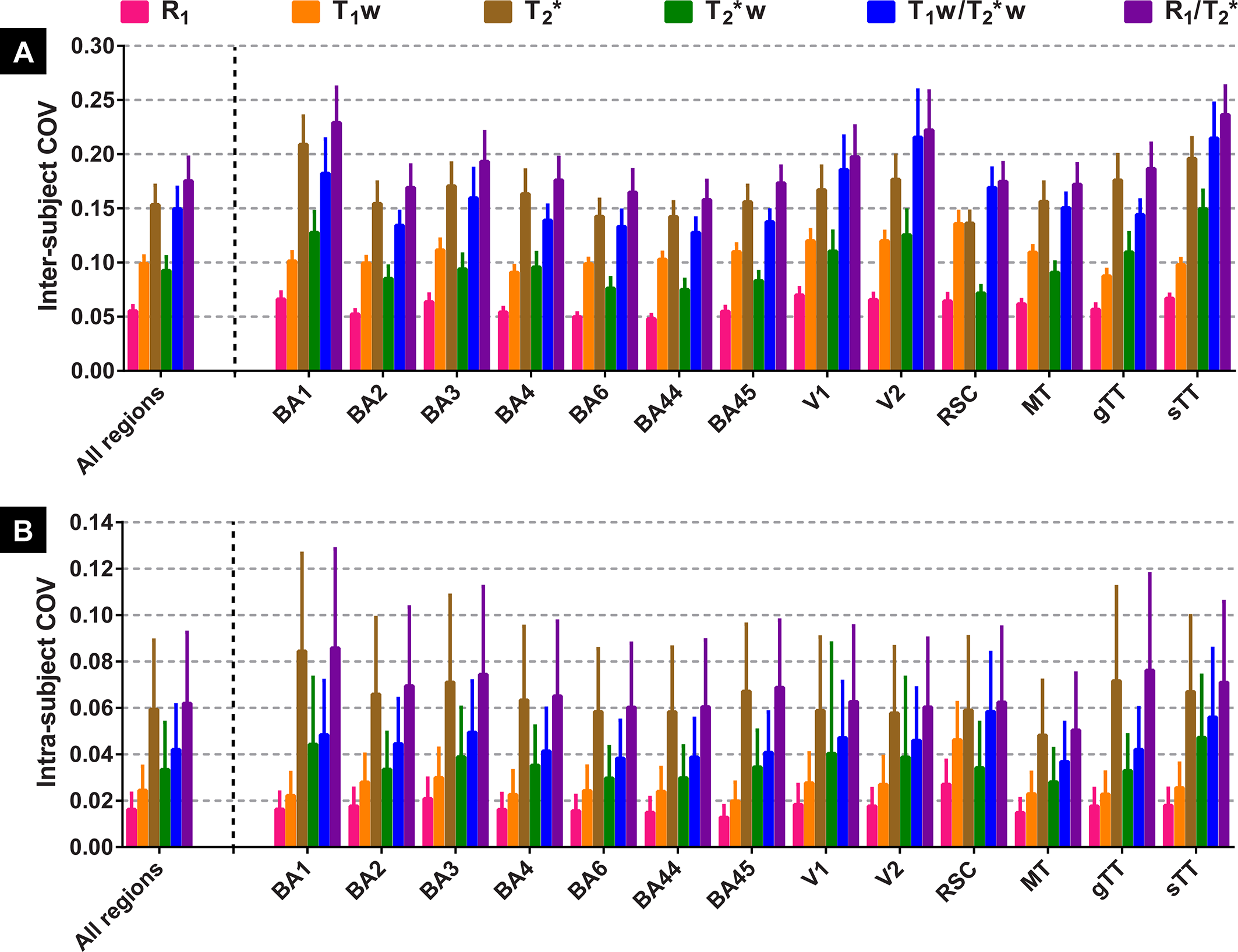

Supplement: Supplementary file 3 [file Image_2.TIF]

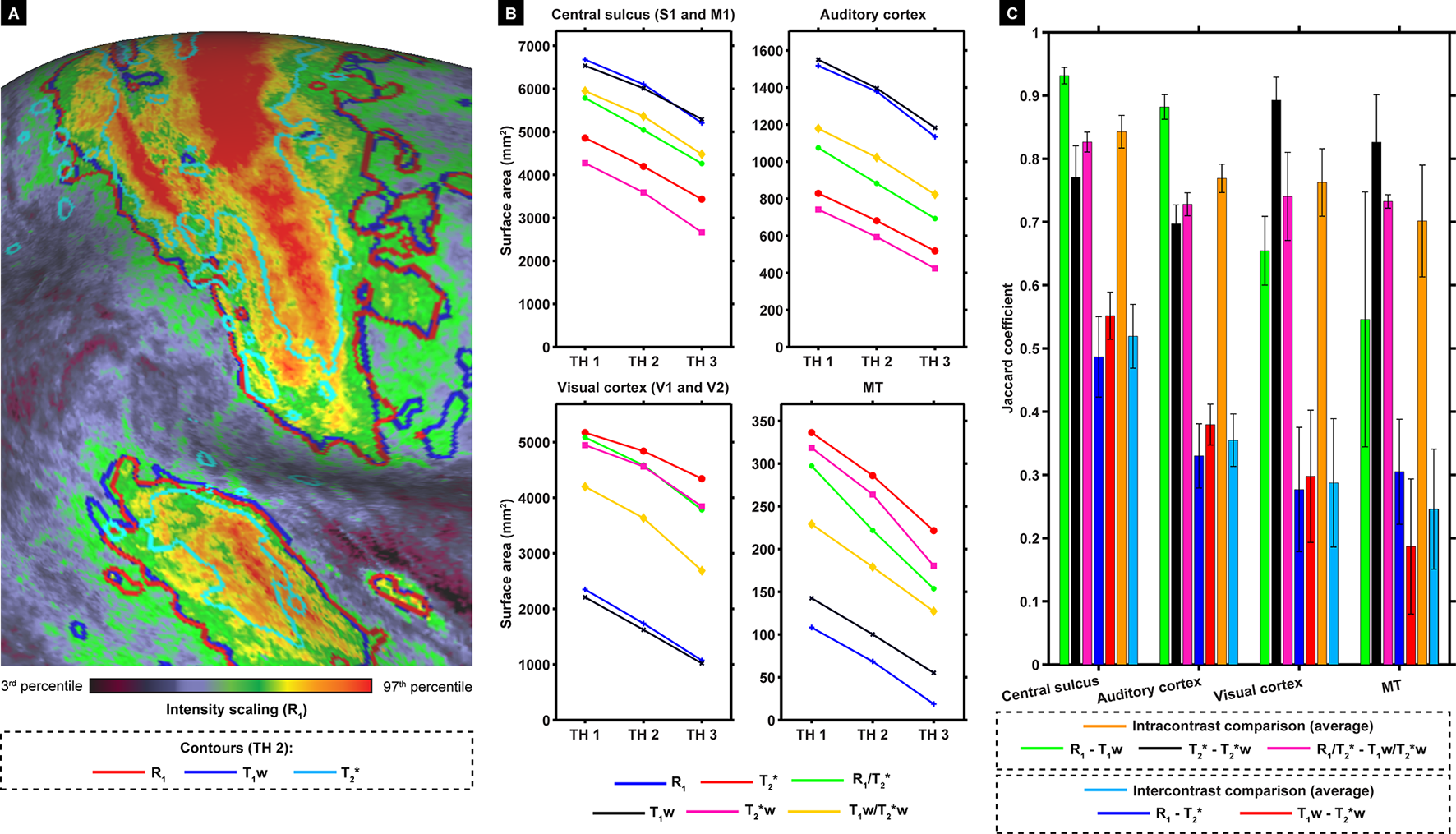

Supplement: Supplementary file 4 [file Image_3.TIF]

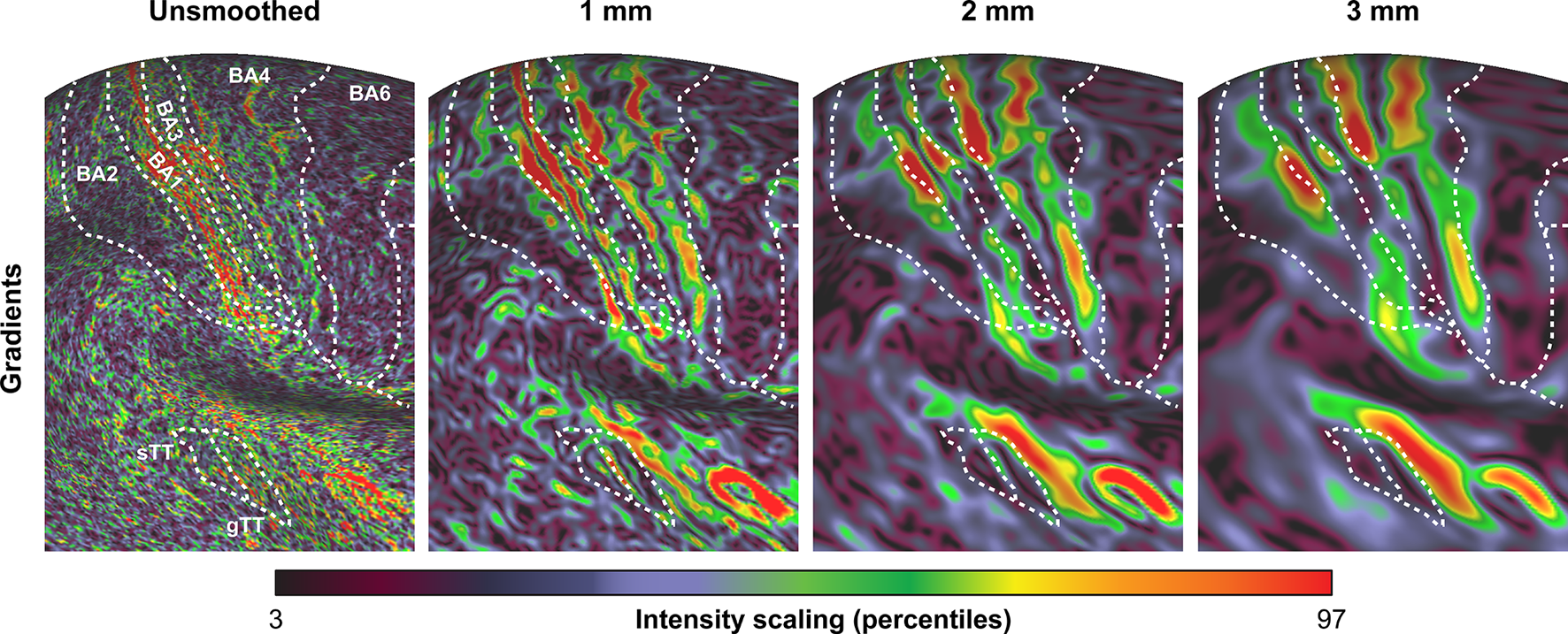

Supplement: Supplementary file 5 [file Image_4.TIF]
